# Towards an ecological definition of sepsis: a viewpoint

**DOI:** 10.1186/s40635-021-00427-2

**Published:** 2021-12-29

**Authors:** Michael Bauer, Manu Shankar-Hari, Daniel O. Thomas-Rüddel, Reinhard Wetzker

**Affiliations:** 1 Department of Anesthesiology and Intensive Care Medicine, Jena University Hospital, Jena, Germany; 2 Center for Sepsis Control and Care, Jena University Hospital, Jena, Germany; 3 Department of Infectious Diseases, School of Immunology and Microbial Sciences, King’s College London, London, UK; 4 Centre for Inflammation Research, University of Edinburgh, Edinburgh, UK; 5 Department of Intensive Care Medicine, Guy’s and St Thomas’ NHS Foundation Trust, London, UK

**Keywords:** Sepsis, Host stress response, Resistance, Tolerance, Energy metabolism, Allostatic overload

## Abstract

In critically ill patients with sepsis, there is a grave lack of effective treatment options to address the illness-defining inappropriate host response. Currently, treatment is limited to source control and supportive care, albeit with imminent approval of immune modulating drugs for COVID-19-associated lung failure the potential of host-directed strategies appears on the horizon. We suggest expanding the concept of sepsis by incorporating infectious stress within the general stress response of the cell to define sepsis as an illness state characterized by allostatic overload and failing adaptive responses along with biotic (pathogen) and abiotic (e.g., malnutrition) environmental stress factors. This would allow conceptualizing the failing organismic responses to pathogens in sepsis with an ancient response pattern depending on the energy state of cells and organs towards other environmental stressors in general. Hence, the present review aims to decipher the heuristic value of a biological definition of sepsis as a failing stress response. These considerations may motivate a better understanding of the processes underlying “host defense failure” on the organismic, organ, cell and molecular levels.

## Introduction

Sepsis is defined as new onset organ dysfunction caused by a dysregulated or failing host response to infection [1], with septic shock reflecting a more severe form [2]. Sepsis is associated with increased risk of short-term morbidity and mortality in intensive care units, as well as long-term sequelae [3]. Incidence, especially in an aging population with comorbidities, remains high. Awareness is also increasing that patients who survive the acute phase often have long-term physical, psychological, and cognitive impairments, albeit the underlying molecular mechanisms remain unclear.

Despite tremendous research efforts over recent decades, no host response-directed therapies for sepsis yet exist. In this context, editorials and review articles often conclude that sepsis is a heterogeneous disease and, to make progress we need to stratify or personalize care. In this review, we propose an alternative hypothesis—*sepsis is an illness state characterized by allostatic overload and failing responses of the organism to infection and other types of environmental stress*.

This line of reasoning is based on similarities in biological phenomena associated with infection-induced sepsis and other causes of critical illness; these are referred to as environmental stressors hereon. A compelling example was provided by Moita and coworkers [4]. They serendipitously discovered suppression of cytokine production by cytotoxic anthracyclines, a chemotherapeutic drug used to treat solid tumors and hematological cancers. In a mouse model of polymicrobial sepsis, animals treated with anthracyclines had a much lower mortality rate compared to controls. Similar effects could be achieved by whole body irradiation [4]. What do cytotoxins and irradiation have in common, and how can they affect immunopathology in sepsis? Both are environmental stressors that induce energy-demanding responses by the affected organism. Physiological responses to stressors appear to compete with the immune system for energy sources and, consequently, the hyperinflammatory processes accompanying sepsis are attenuated. This interpretation leads to the hypothesis that stressing the energy source—for example by food deprivation—could provoke similar effects on sepsis progression. Indeed, dietary restriction, highly significant evolutionary stressor, does restrain hyperinflammatory responses in septic mice, especially if fasting commenced prior to the onset of sepsis [5].

Based on these and other observations, a close relationship between sepsis aetiopathology and general environmental stress responses becomes evident. The failing responses to infectious pathogens in sepsis are intimately interwoven with the organism’s reaction to other environmental stressors, emphasizing an ecological dimension to the disease.

The present review explores the empirical benefit of considering a biological definition of *sepsis as a failing stress response*. Embedding pathogen-induced reactions within the ancient responses of organisms to environmental stress in general, will broaden the understanding of sepsis pathobiology and generate novel targets.

### Effective and failing responses to environmental stressors

**Fig. 1:**
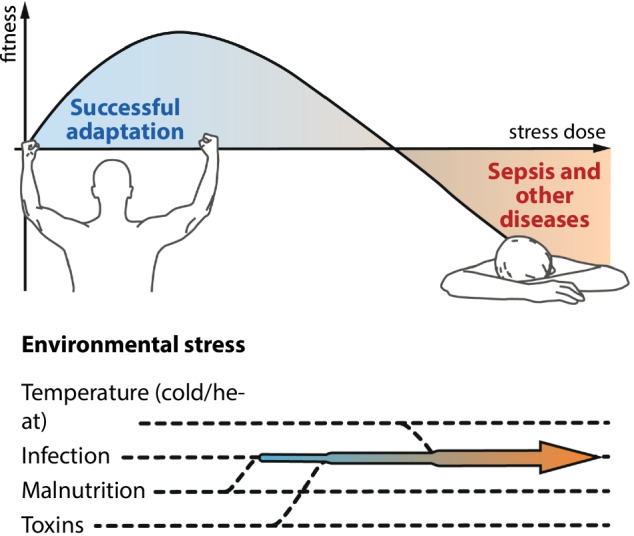
Adaptive and maladaptive responses to environmental stress

In sepsis, biotic (pathogens) and abiotic (environmental) stressors cause sepsis-related organ dysfunction through concurrent functional impairment of cells without significant structural damage [6]. The resulting patient host response could be either adaptive or maladaptive (Fig. 1). Abiotic stressors include mechanical stress (injuries, training), deviant outside temperature and food restriction among others.

Each organism exhibits a threshold beyond which successful responses to a certain dose of a specific stressor turn to increasingly failing responses and impaired fitness (disease). The impact of stress on the affected organism thus depends on the dose, i.e., intensity, of the stress event.

A simple example is the limited ability of organisms to tolerate cold or heat. Both excessive cold or heat to a specific body region leads to damage of affected organs and tissues.

Similarly, in experimental sepsis research a well-controlled increase in pathogen dose is reflected by altered mortality rates [7]. Soluble pathogen-associated molecular patterns (PAMPs) and dead microorganisms pose a relatively low-level threat to the host as compared to viable microorganisms. The threat of viable microorganisms depends on both direct and indirect effects of virulence factors. This is partly replicated in animal models: For instance, in a peritoneal contamination and infection (PCI) rodent model, increasing injection of stool suspension induces a nonlinear rise of mortality [8]. Whereas a low pathogen burden induces an efficient series of responses, increasing doses are associated with failing response patterns with increased mortality. These findings highlight the central role of the intensity of infectious stress in the development of the disease process.

Thus, the conceptual model of sepsis we are proposing here is a maladaptive host response to infectious stress that is influenced by both abiotic environmental stress and the patient’s baseline health. Modulation of the maladaptive host response by environmental stress can lead to both improvement or deterioration of the patient’s condition. The latter setting can be defined as “allostatic overload”, a term created by McEwen and Stellar in 1993 to depict pathophysiological consequences of repeated or prolonged stress [9]. These principles seem highly evolutionary conserved and are thus reproducible across species despite all limitations of mouse models of sepsis.

Ahead of considering maladaptive responses of the organism in sepsis, we will first summarize physiological mechanisms that commonly protect against the harmful effects of invading pathogens and of other environmental stressors.

### Countering infection- and environment-related stress

In general, organisms counter environmental stress, including infection, through three distinct strategies: avoidance, resistance, and tolerance [10]. For example, humans exposed to cold conditions can respond by insulation of the body core from the skin, by increasing metabolic heat production or by tolerating core hypothermia. The type of response is dependent on energy intake, the amount of insulating body fat and inherited or acquired adaptation [11]. Resistance and tolerance responses as complementary concepts similarly shape human responses to microbial invasion of normally sterile tissue compartments. If infectious stress cannot be avoided, resistance reactions of the immune system are activated to destroy the invading microorganisms. The multifaceted *resistance* responses of the innate and adaptive immune systems share one general feature, namely a large energy requirement. Consequently, energy resources are limited, necessitating a shift of balance towards more energy-saving or efficient immune and other host responses. The resultant reaction pattern is referred to as tolerance to environmental stress, however its relevance to infectious stress responses has only been realized in the last decade [10, 12]. With tolerance to infection, molecular and cellular attacks on the invading microorganism are reduced; cellular maintenance reactions will be initiated to passively overcome the damaging effects of pathogens [13, 14]. These maintenance responses can include depletion or repair of cells and tissue damaged by pathogens, or by an excessive pro-inflammatory response.

Resistance and tolerance responses are however spatially and temporally interwoven, with shifts in balance in either direction dependent on the energy status of vital cells involved. It could be conceptualized that the pathogen response is intimately embedded with the energy status of the organism, and also affected by concomitant challenges through other environmental stressors. Both resistance and tolerance responses of the organism to microbial infections involve not only the immune system, but also cells (and their organelles) within solid organs such as liver and kidneys [14].

How does the pathogen dose affect immune resistance and tolerance? The inflammatory response of innate immune cells depends strongly on the strength and duration of exposure to the bacterial endotoxin, lipopolysaccharide (LPS) [15]. Enhanced release of pro-inflammatory mediators is detected in monocytes after priming with low doses of LPS, while suppression occurs with higher doses [16]. A shift from an enhanced resistance response to immune tolerance was also observed in vivo after treating mice with increasing doses of LPS [17]. These in vitro and in vivo investigations also highlight the key role of pathogen dose in the progression of resistance reactions to maintenance and repair responses in predominantly tolerant cells and organs [13].

Adaption of body temperature in response to infection also provides another intriguing example of the close interrelation between resistance and tolerance responses of the immune system and other key physiological functions. Associations are seen between immune resistance and fever, as well as immune tolerance and hypothermia. Both complementary organismic response patterns appear linked to pathogen dose. The impact of increasing doses of LPS on body temperature in rats was investigated by Romanovsky et al. as far back as 1996 [18]. This study found significant increases of colonic temperature with LPS doses up to 100 μg/kg. In marked contrast, injection of 1000 μg/kg induced a strong decrease in body temperature. The authors interpreted this shift from fever to hypothermia as a sign of the change in balance from resistance to tolerance, combined with an accelerating metabolic exhaustion of the organism [19].

**Fig. 2:**
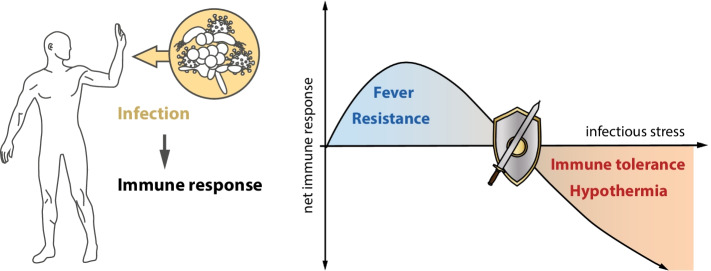
Complementary patterns of immune responses and thermoregulation to infection. Key role of pathogen-induced infectious stress dose

Taken together, these studies indicate close similarities in the response pattern of fever and immune resistance reactions as well as hypothermia and immune tolerance, to infectious stress (Fig. 2). Both thermoregulatory responses and immune-driven inflammatory reactions are strongly influenced by pathogen-induced infectious stress where the pathogen load shapes the host’s adaptive response to infection.

Acting like a sword and shield all higher organisms have evolved two complementary strategies to overcome attack by invading microbes: namely resistance and tolerance. Resistance responses, primarily mediated by specialized immune cells, aim to destroy the pathogen. Tolerance does not affect pathogens directly, but initiates maintenance and repair reactions to prevent damage to parenchymal tissue. Resistance and tolerance responses do not proceed one by one, but are intimately interwoven both in time and co-location. After a pathogen attack, resistance reactions frequently predominate, followed by a shift of balance towards tolerance responses secondary to metabolic deficiencies. The pathogen response is closely embedded within the energy status of the organism and is also affected by challenges of other stressors such as heat and cold. As a rule, pathogen load significantly impacts upon progression of the response pattern, boosting tolerance responses at high doses. Hence, low pathogen doses induce an anabolic resistance response, whereas high doses induce energy-saving catabolic and maintenance responses, such as autophagy.

Evidence for a competition of physiological response to different stressors including infection, cold and malnutrition was recently reported by Ganeshan et al. [20]. Energetic trade-offs were seen between immunity, homeothermy and hypometabolic states, which promote disease tolerance during bacterial infections. These findings support our proposition regarding interwoven stress responses by the infected host.

This response pattern, which is also valid for other stress types (“stress and strain”), is reversible under physiological conditions, restoring homeostasis. In sepsis, however, both mechanisms to react to invading pathogens and are apparently out of control.

### Molecular signatures of complementary immune responses

How do cells sense and process different doses of pathogens? As mentioned above, increasing pathogen-induced infectious stress is accompanied by energy exhaustion. Energy-demanding anabolic resistance responses deplete ATP and increase intracellular levels of ADP and AMP. This metabolic shift provokes stimulation of the crucial intracellular energy sensor, AMP-activated protein kinase (AMPK). AMPK induces reprogramming of cellular metabolism from an energy-consuming anabolism to catabolic and regenerative processes [21]. As a result, AMPK maintains the energy balance by decreasing synthesis of proteins, fatty acids and other biomolecules, while regenerative processes, including autophagy and mitochondrial biogenesis, are increased. The stringent inhibitory effects of AMPK on anabolic processes are mediated via suppression of the signaling activity of mTOR (mechanistic target of rapamycin). mTOR is a key mediator of protein synthesis, cell growth, and other ATP-consuming processes, including immune resistance responses [22].

**Fig. 3:**
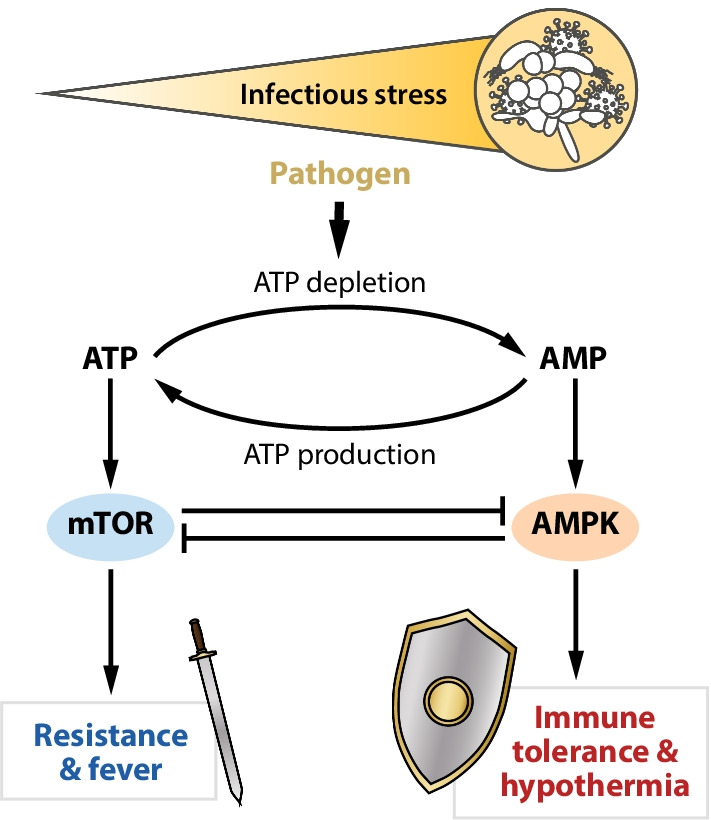
Dose-dependent effects of pathogens on energy metabolism provoke complementary immune- and thermoregulatory responses to infection

In the context of pathogen-induced energy depletion, Fig. 3 depicts a tentative overview of the involvement of AMPK and mTOR in metabolic control of immune responses and temperature regulation. Whereas the mTOR signaling pathway plays a prominent role in resistance reactions of immune cells, AMPK is a key mediator of maintenance and repair responses leading to tolerance. Resistance reactions frequently associated with fever, and immune tolerance linked to hypothermia, may represent consecutive steps of the response pattern of immune cells to increasing doses of PAMPs. The mutual ability of mTOR and AMPK to inhibit each other [13] complies with the proposed key functions of these mediators in the adjustment of antagonistic resistance or tolerance responses.

The close links of resistance responses to fever, and of immune tolerance to hypothermia, suggests crucial signaling mediators of thermoregulation. Fever as the “attendant” of immune resistance is initially driven by anabolic processes such as cytokine production, which require functional mTOR. By contrast, with cellular and organismic energy exhaustion and accompanying hypothermia, AMPK takes over control with catabolic maintenance and subsequent repair processes [21, 22].

The identification of mTOR and AMPK as central mediators of immune resistance and tolerance responses and their proposed role in thermoregulation integrates pathogen-induced reactions into an ancient response pattern of cells and organs to other environmental stressors. In the context of thermoregulation, dose-related effects of ambient temperature on the host’s thermoregulatory and immune responses can be anticipated. The energy demands of the adaptive response, i.e., dose-dependent effects of all prevailing environmental stressors on cellular metabolism, will determine specific responses and the fate of affected cells, organs and organism [13].

To survive, cells, organs and organisms must adapt. Adaptation is aimed at restoring homeostasis and maintaining the fitness of the host when affected by pathogen attacks and other environmental stressors.

### Sepsis as a failing stress response

Successful adaptation to environmental microbes is a daily routine for all higher organisms. Both resistance and tolerance responses contribute to maintenance of health and a fast, efficient recovery. In serious infectious diseases, and specifically in sepsis, the ability to restore homeostasis is effectively lost. Failing responses to pathogen stress are hallmarks of disease progression. Understanding of the transition from successful to failing response patterns becomes a pivotal issue of sepsis research.

**Fig. 4:**
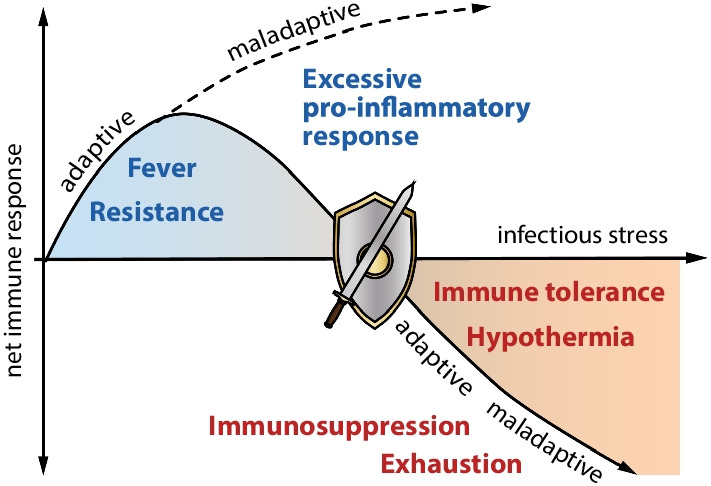
Failing complementary immune responses and thermoregulation in sepsis

Sepsis was long interpreted as an overwhelming systemic inflammatory response to infection [1]. Pathogen-induced uncontrolled immune responses were considered causative of impaired organ function. However, the exclusive focus on systemic inflammation was challenged by the finding that a significant proportion of septic patients exhibit symptoms of immune suppression (and hypothermia). Reduced responsiveness of innate and adaptive immune cells (immunosuppression) dominate sepsis pathology, at least in later stages of the disease [23, 24]. Both, excessive pro-inflammatory responses as well as immunosuppression, can provoke remote organ failure (Fig. 4).

With progression of sepsis and (multi-) organ dysfunction reversibility of resistance and tolerance responses towards invading pathogens is lost. Many patients initially exhibit vigorous inflammatory reactions, which increasingly damage host tissue. Molecules released by damaged cells promote futile inflammatory processes in an auto-accelerating manner leading to further impairment and, lastly, to metabolic exhaustion of inflamed tissues. Patients surviving the excessive pro-inflammatory episode frequently lapse into a state of prolonged immunosuppression. In contrast to tolerance responses these processes do not lead to homeostasis but pave the way for long-term impairment of immune and parenchymal functions. Progression of dysregulated inflammation and immunosuppression may also depend upon environmental conditions such as temperature and nutritional status.

Why some infected patients develop excessive pro-inflammatory responses and immunosuppression is still largely unknown. Following the concept of functionally complementary responses to microbial infection, the similarities of excessive pro-inflammatory responses and immunosuppression to the phenomena of immune resistance and tolerance become strikingly evident.

Resistance responses aim at attacking and destroying invading pathogens, e.g. cocktails of proteases and reactive oxygen species, can also damage host cells near the site of infection. Consequently, excessive resistance responses may cause collateral tissue damage in close correlation to the local intensity.

Immunosuppression, frequently observed during the course of sepsis, shares core characteristics with immune tolerance. Pathogen-directed resistance responses are attenuated or shut down. However, whereas in tolerant organisms the balance between damaging processes induced by the invading microorganism and concomitant regenerative processes in the host remains well-adjusted, during immunosuppression disseminating microorganisms may provoke escalating tissue damage. Consequently, septic immunosuppression with unfettered opportunity for pathogens to spread and cause damage must be separated from disease tolerance albeit supporting data for immunosuppression as dysregulated “tolerance” are scarce. Consistent with our proposed concept, disseminating pathogens and malfunctioning regenerative processes will impair host parenchyma and organ function (Fig. 4).

What about the role of pathogen load in the pathophysiology of sepsis? As mentioned above, a critical microbial load is a prerequisite for causing mortality in sepsis models. However, surprisingly, there are few mechanistic and clinical investigations examining the relationship between pathogen load and propagation of organ dysfunction. Initial insights into the impact of microbial load on the course of sepsis are provided by investigations into the balance between two established cytokine biomarkers of immunopathology and immunosuppression. Whereas sepsis patients exhibiting low bacterial load predominantly release the cytokine, interferon-gamma (IFNγ), the inflammation-inhibiting cytokine, interleukin-10 (IL-10), prevails in bacteremic patients with a high bacterial load [25]. Recently, König et al. mimicked the bacterial burden on the immune system in sepsis by challenging human volunteer blood with increasing doses of bacteria [26]. Interestingly, both IFNγ and IL-10 increased with rising bacterial challenge, but the ratio showed the opposite behavior, i.e., a high bacterial load was associated with a distinct overbalance of the anti-inflammatory cytokine IL-10. Notably, comparable data on pathogen dose effects have been obtained for viral infection with SARS-CoV-2 viral load associated with increasing disease severity and mortality [27].

The predominance of either excessive pro-inflammatory responses or immunosuppression in sepsis also seems to be associated with two opposing states of thermoregulation. In a recent study, Thomas-Rüddel et al. determined two subsets of septic patients, one with pronounced fever and another with hypothermia [28]. These phenotypes where associated with environmental temperature lending support to our assumption of a link between energy expenditure and resistance or disease tolerance, respectively [29]. Interestingly, the hypothermia group had the highest mortality rates. Cytokine responses and other clinical parameters clearly characterize the close relationship of fever patients to the pro-inflammatory phenotype, whereas hypothermia is preferentially observed in patients exhibiting a significant decrease in cytokine response, which is a main characteristic of immunosuppression. Thus, the close relationship of hypothermia to the tolerance phenotype also appears in septic patients presenting with signs of immunosuppression. A similar link seems to be valid for fever and resistance responses of different intensity including excessive inflammation in sepsis. Together these findings support the concept of resistance and tolerance as closely connected complementary response patterns to infection, as well as in the failing host response to infection that characterizes sepsis.

### Derived biological concepts to treat sepsis

**Fig. 5:**
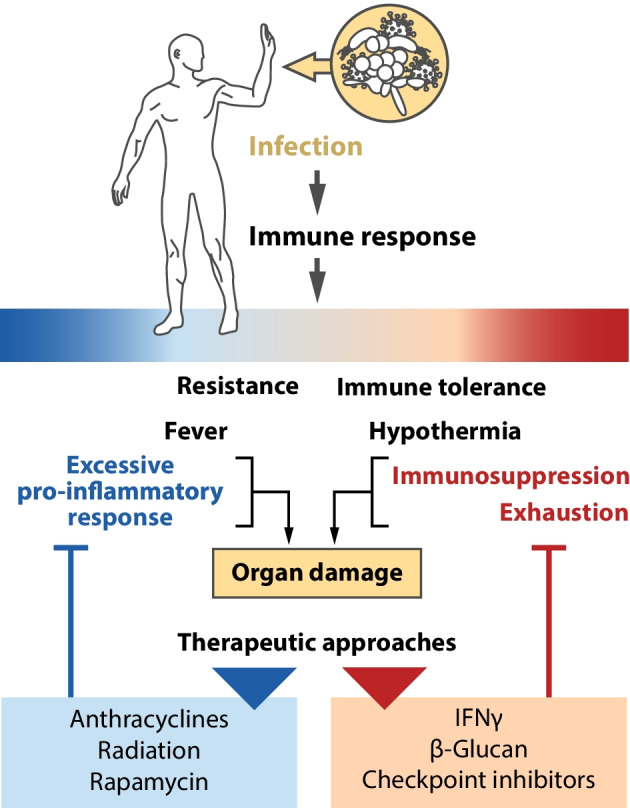
Evolving therapeutic concepts to affect failing resistance and tolerance responses to infectious stress in sepsis

These insights into the etiology of sepsis could be used to direct the development of novel treatment approaches (Fig. 5).

In sepsis overwhelming resistance responses provoke tissue damage, whereas excessive tolerance might cause immunosuppression and devastating infection. Embedding these failing reactions in response patterns of the organism to other stressors, treatment of these complementary inappropriate immune states in well-designed mouse models seems possible by induction of the alternative response. At least in animal models of sepsis, tissue damage induced by overflowing resistance responses have been successfully treated by triggering tolerance responses, whereas tolerance-induced immunosuppression has been successfully treated by provoking resistance reactions in innate immune cells. All these innovative treatment ideas have been achieved by application of stressors unrelated to the microbial pathogens, such as anthracyclines or radiation. Chronic inflammatory diseases, characterized by enduring immunogenic resistance reactions and damage of parenchymal cells, can be successfully treated by cold, heat, radiation or other stressors. Thus, resistance and tolerance responses to pathogens appear to be embedded in the ecological reaction pattern of the organism, allowing specific targeting of malfunctioning immune responses by seemingly unrelated environmental stressors.

In addition to the initially mentioned application of anthracyclines, irradiation and food deprivation [4, 5], excessive pro-inflammatory reactions and fever accompanying dysregulated resistance responses in rodent sepsis can be inhibited by the immunosuppressive and antiproliferative agent rapamycin, which is the eponymous inhibitor of mTOR [30]. As per Fig. 3, one might predict suppressive effects of the natural mTOR antagonist AMPK on the excessive pro-inflammatory response seen in sepsis. Indeed, pharmacological stimulation of AMPK by metformin protected against sepsis-induced organ injury and inflammation [31]. The limitations of such models does have to be considered. In particular, murine sepsis models do not reflect human metabolic responses to infectious and other stressors, thus revealing risks for the translation of these preclinical studies into successful clinical trials [20, 32]. However, the findings do reveal energy-consuming responses to environmental stressors as well as inhibitors of anabolic processes as key candidates for innovative treatment prospects for patients with an exaggerated pro-inflammatory response and concomitant organ damage. Given the key role of dose response characteristics in the presented hypothesis, one might assume that a theranostic biomarker might be required to identify the more severe cases in which the proposed interventions into these master regulators of energy metabolism might be therapeutically promising.

On the other hand, sepsis-induced immunosuppression, which can be interpreted as a dysregulated immune tolerance, can be efficiently counteracted by immunostimulatory mediators, such as growth factors or cytokines [33]. Partial reversion of immunosuppression in humans has been shown by treatment with IFNγ [33]. Immunostimulatory agents used in preclinical models cover a broad spectrum of agents including the pathogenic yeast *Candida albicans* and its cell wall component β-glucan [34]. Furthermore, checkpoint inhibitors, which can increase immune cell proliferation and concomitant cytokine production could be used to compensate immunosuppression [35]. However, all these approaches will be reliant on improved patient stratification. Patients exhibiting characteristics of immunopathology can already be discriminated from patients with immunosuppression. Systemic parameters, such as immune status, body temperature and molecular response patterns of impaired organs, could be used to find the most efficient decisions for the treatment of these contrasting dysfunctional states of the immune system.

## Synopsis and conclusions

Definitions of sepsis currently focus on pathology, clinical management, and epidemiology of the disease. Hence, the current definition, Sepsis 3, [1] defines sepsis as a life-threatening organ dysfunction caused by a dysregulated host response to infection. For clinical operationalization, organ dysfunction has been defined by the Sequential Organ Failure Assessment (SOFA) score, which is associated with in-hospital mortality. This highlights the role of the host response, but deciphering the sepsis-related physiological and pathological phenomena in living nature are beyond its scope.

Our interpretation of sepsis tries to focus on the relationships of the disease to these phenomena. We propose that sepsis be viewed as a complication of infectious disease caused by a malfunctioning stress response, for which the interplay between temperature responses and microbial infections serve as an example, including their specific effects at the organismic, organ-specific, cellular and molecular level. This alternative perspective may help to identify novel treatment options for sepsis and other severe infectious diseases.

Collectively, the suggested designation of sepsis as an example of failing adaptive responses of higher organisms toward environmental stressors could inspire and motivate future collaborations of clinician scientists and basic researchers for joint experimental efforts to understand and combat sepsis.

### Open questions/perspectives

The loss of balance of resistance and tolerance responses to infectious attacks should be considered a core concern in sepsis research. How does resistance goes out of control and moves to an excessive pro-inflammatory response, and how do impaired tolerance reactions shift to immunosuppression, are currently poorly understood. To understand this enigma, we suggest to broaden the view and to embed the catastrophic progression of an infection to sepsis in the general stress response pattern of the organism. Appreciating the role of environmental stressors such as heat, cold, (mal-)nutrition, toxins and radiation may enable deeper insights into the mechanisms of sepsis and, in a best case scenario, novel and effective therapeutic approaches.

## Data Availability

Not applicable.

## Glossary

AMPK (AMP-activated protein kinase): Key sensor and mediator of energy expenditure and homeostasis in cells.
DAMPs (damage-associated molecular patterns): Intracellular molecules released by activated or necrotic cells following tissue damage.
β-Glucan: Cell wall component of *Candida albicans*.
Hormesis: Biphasic dose response phenomenon characterized by low-dose stimulation and high-dose reduction of vitality parameters.
LPS (lipopolysaccharide): Cell wall component of Gram-negative bacteria.
Maintenance: Umbrella term for intracellular processes aimed at the degradation of malfunctioning molecules and organelles and restoration of homeostasis.
mTOR (mechanistic target of rapamycin): Key mediator of anabolic processes in cells.
PAMPs (pathogen-associated molecular patterns): Cell components of pathogenic bacteria and fungi that signal the threat of infection to affected cells and organisms.
Resistance: Immune responses that reduce pathogen burden after infection.
Tolerance: Responses of immune and parenchymal cells that reduce the negative impact of an infection on host fitness without directly affecting pathogen burden.
